# Supplementary material for: Comparative multiomics analysis of cell physiological state after culture in a basket bioreactor
Source: Sci Rep. 2022 Nov 23;12:20161. doi: 10.1038/s41598-022-24687-4 (PMC9686226; doi:10.1038/s41598-022-24687-4)

23666033.1:19011265:19014725:-@NW_023666033.1:19008079:19014725:-@NW_023666033.1:19008079:1900[illegible]

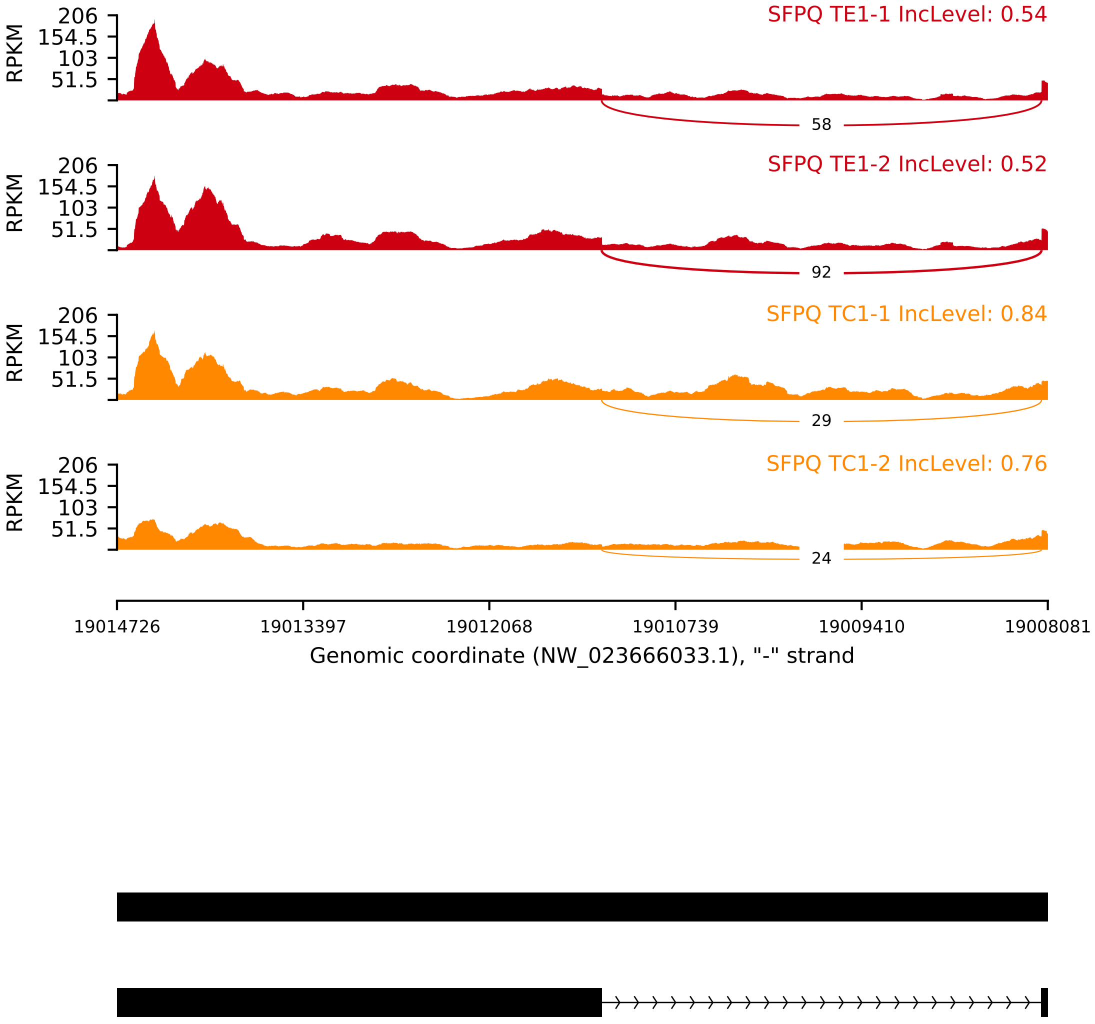

Supplement: Supplementary file 1 — Supplementary Information 1. [file 41598_2022_24687_MOESM1_ESM.zip › raw data/Transcriptomic raw data/7.AS/2.dasplot/TE1vsTC1/RI/5_SFPQ_NW_023666033.1_19011265_19014725_-@NW_023666033.1_19008079_19014725_-@NW_023666033.1_19008079_19008126_-.pdf]
